# Extraskeletal myxoid chondrosarcoma of the gingival: a rare case report and review of the literature

**DOI:** 10.1186/s13000-023-01390-0

**Published:** 2023-09-13

**Authors:** Jiaqi Li, Zhijian Zheng, Hao Deng, Yi Men, Yu Chen, Qi Han

**Affiliations:** 1 State Key Laboratory of Oral Diseases &, National Clinical Research Center for Oral Diseases, West China Hospital of Stomatology, Sichuan University, Chengdu, 610041 P.R. China; 2 State Key Laboratory of Oral Diseases &, Department of Oral Pathology, National Clinical Research Center for Oral Diseases, West China Hospital of Stomatology, Sichuan University, Chengdu, 610041 P.R. China; 3 Department of Stomatology, The First People’s Hospital of Ziyang, Ziyang, 641300 P.R. China; 4 State Key Laboratory of Oral Diseases &, Department of Head and Neck Oncology, National Clinical Research Center for Oral Diseases, West China Hospital of Stomatology, Sichuan University, Chengdu, 610041 P.R. China

**Keywords:** Extraskeletal myxoid chondrosarcoma, Malignant tumor, Mandibular gingiva, Immunohistochemistry, NR4A3, Differential diagnoses

## Abstract

**Background:**

Extraskeletal myxoid chondrosarcoma (EMC) is a rare malignant tumor described in the head and neck region, especially in the gingival. We present one case arising in the gingival of right mandible, and briefly reviewed the related literature.

**Case presentation:**

A 24-year-old male patient with a lesion of 3.5*2.0 cm in buccal gingival of right posterior mandible for 2 months. The tumor was composed of cartilaginous structures and myxoid matrix. Immunohistochemical(IHC) showed that the tumor cells to be positive for vimentin, focally positive for S-100, negative for calponin, SMA, SOX10. The Ki-67 labelling index was 80%. Fluorescent in situ Hybridization (FISH) was positive for NR4A3 rearrangement.

**Conclusions:**

Due to its unusual site and low incidence in the oral region, a combination of histological findings, immunohistochemistry, and molecular pathology as well as differential diagnosis with other diseases should be taken into consideration in the process of clinical diagnosis and treatment.

## Introduction

Extraskeletal myxoid chondrosarcoma(EMC) is a rare malignant tumor accounting for less than 3% of all soft-tissue sarcomas [1], and mainly affects the lower limbs of male patients in their fifth and sixth decades of life, clinical signs and symptoms are mostly nonspecific [1, 2]. EMC does not present convincing evidence of cartilaginous differentiation and recently, a neuroectodermic origin has been proposed [3]. Microscopically, the tumor cells of EMC typically present eosinophilic granular, frequently vacuolated cytoplasm with round to oval nuclei, morphologically resembling lipoblasts, immersed in a myxoid stroma in a multilobular arrangement, which is separated by fibrous septae [1]. The immunophenotype of EMC include positivity for vimentin and variable staining for S-100 and neuroendocrine markers Fluorescent in situ Hybridization (FISH) reveals mostly EWSR1-NR4A3 or TAF15-NR4A3 fusion [4].

## Case report

A 24-year-old male patient with a 2-month history of a mass in buccal gingival of posterior mandible. The mass was about the size of a soybean and extended to the lingual side of the mandible, tough and hard in texture, with no significant clinical complaints, such as bleeding or numbness. After the biopsy, the tumor continued to enlarge rapidly and was accompanied with numbness and tenderness. The pathological results of the biopsy were low grade chondrosarcoma of osteogenic origin.

**Fig. 1:**
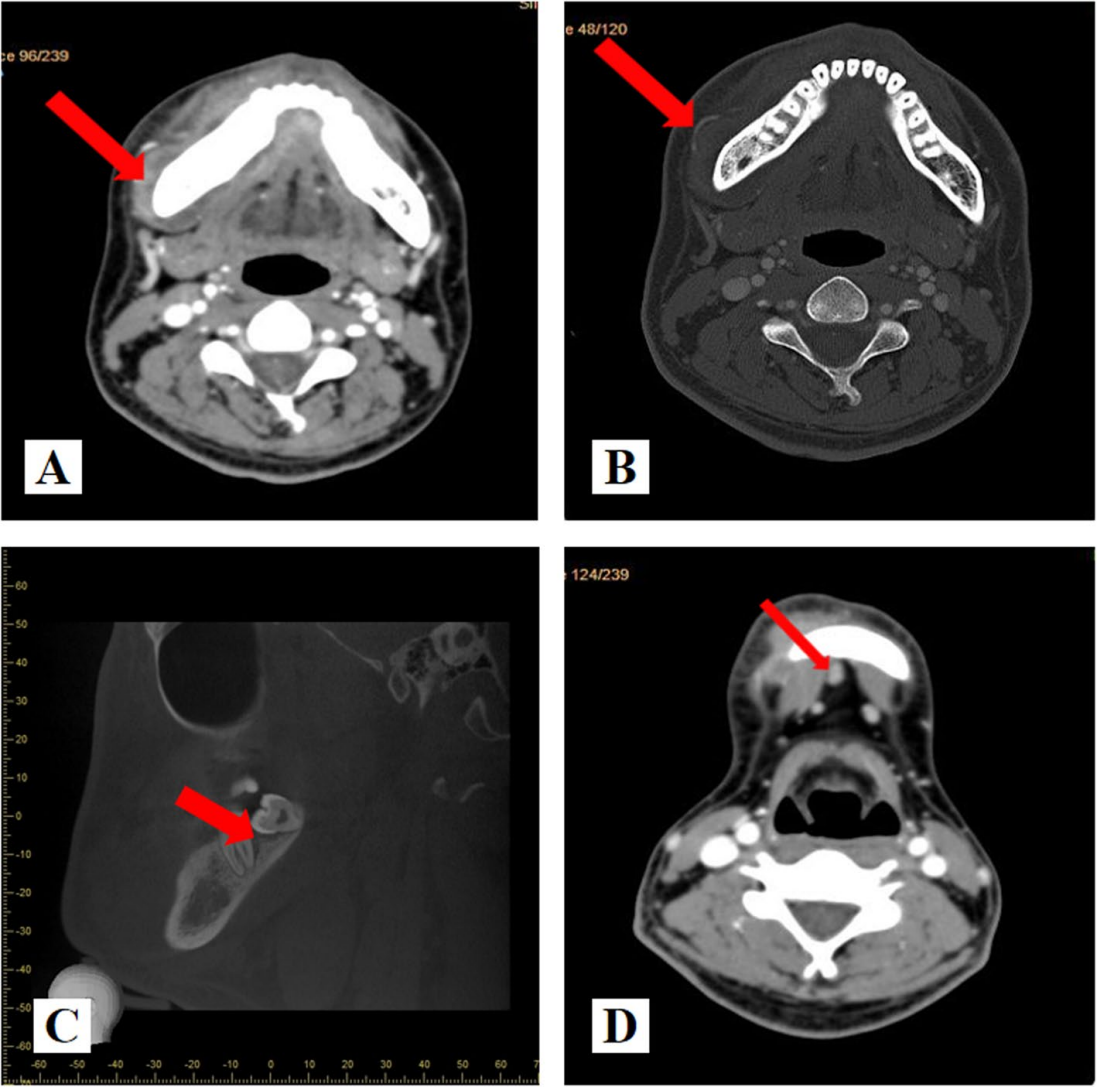
Imaging manifestations of EMC. **A** soft tissues of the labial buccal and lingual side of the right mandible were irregularly thickened(arrow). **B** There appeared to be irregular periosteal reaction in the lateral part of the right mandible(arrow). **C** periodontal space broading were observed in the 47 distal and 48 teeth(arrow).(D)There was no obvious enlargement of submental lymph nodes(arrow)

Enhanced spiral Computed Tomography(CT) combined with Cone Beam Computed Tomography (CBCT) confirmed that the soft tissues of the labial buccal and lingual side of the right mandible were irregularly thickened, spanning the midline and reaching as far as the labial side of the right mandibular cuspid (Fig. 1A and B). There appeared to be irregular periosteal reaction in the lateral part of the left mandible (Fig. 1B), periodontal gap widening as well as small amount of irregular osteoid hyperplasia at the alveolar crest were observed to the right mandibular molar region (Fig. 1C). Submental lymph nodes that are normal in size (Fig. 1D).

The patient underwent extensive resection of the sarcoma of the right mandible and segmental resection of the right mandible. Four months after surgery, recurrence occurred.

**Fig. 2:**
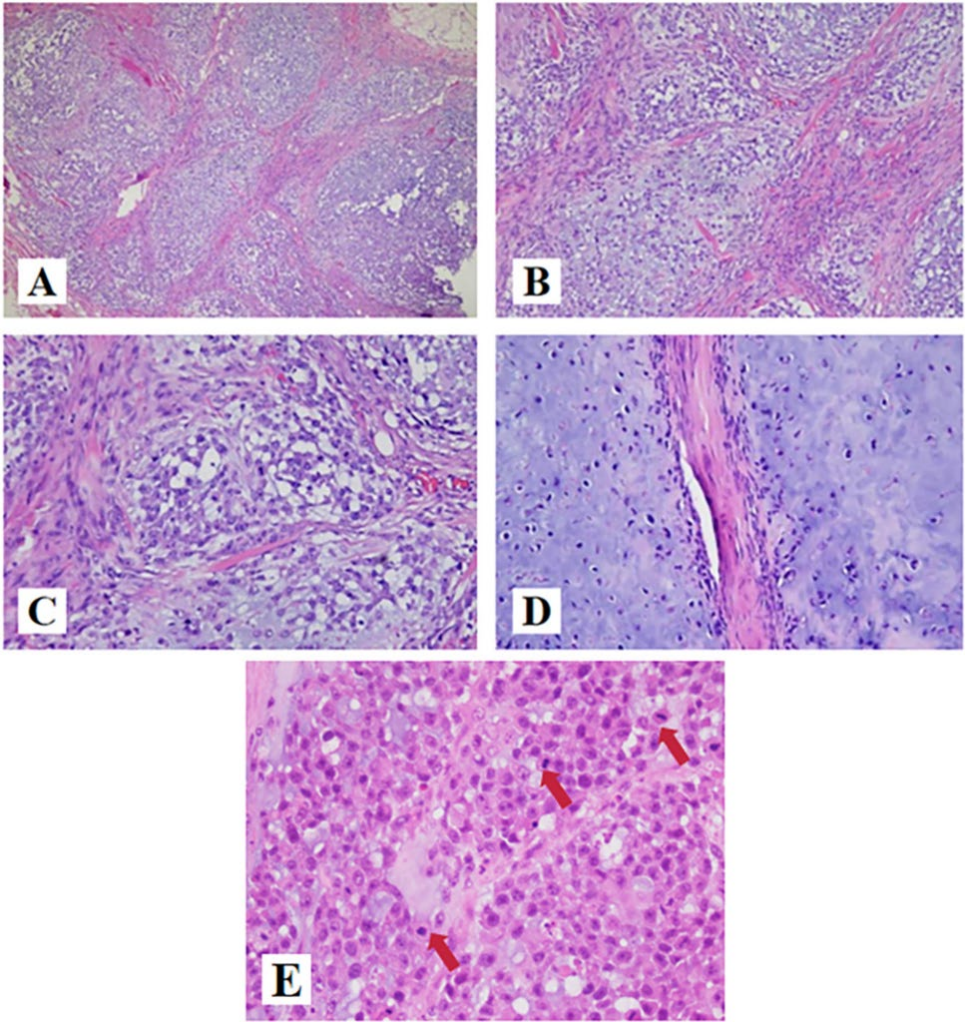
H&E findings of EMC. **A** (HE × 40): the tumor cells immersed in a myxoid stroma in a multilobular arrangement, which is separated by fibrous septae. **B** (HE × 100): The cells were arranged in ribbons, trabeculae and small nests present in a myxoid background. **C** (HE × 200): At high magnification, the tumor cells were uniform in size, with round and oval nuclei, deep chromatin and large nuclear atypia. **D** (HE × 200): The tumor cells were located in the cartilage lacunae, showing atypia and mitosis. **E** (HE × 400): Mitosis was obviously(arrow)

The haematoxylin and eosin revealed that tumors cellswere round or slightly elongated of uniform shape and size with avariable amounts of mucoid matrix in a multinodular arrangement, and the tumor lesion presented a multinodular and lobular pattern with fibrous septa (Fig. 2A), The cells were arranged in ribbons, trabeculae and small nests present in a myxoid background (Fig. 2B). At high magnification, the tumor cells were uniform in size, with round and oval nuclei, deep chromatin and large nuclear atypia (Fig. 2C). The tumor cells were located in the cartilage lacunae, showing atypia and mitosis (Fig. 2D). Mitotic figures are about 3/10 high-power fields (HPFs) (Fig. 2E).

**Fig. 3:**
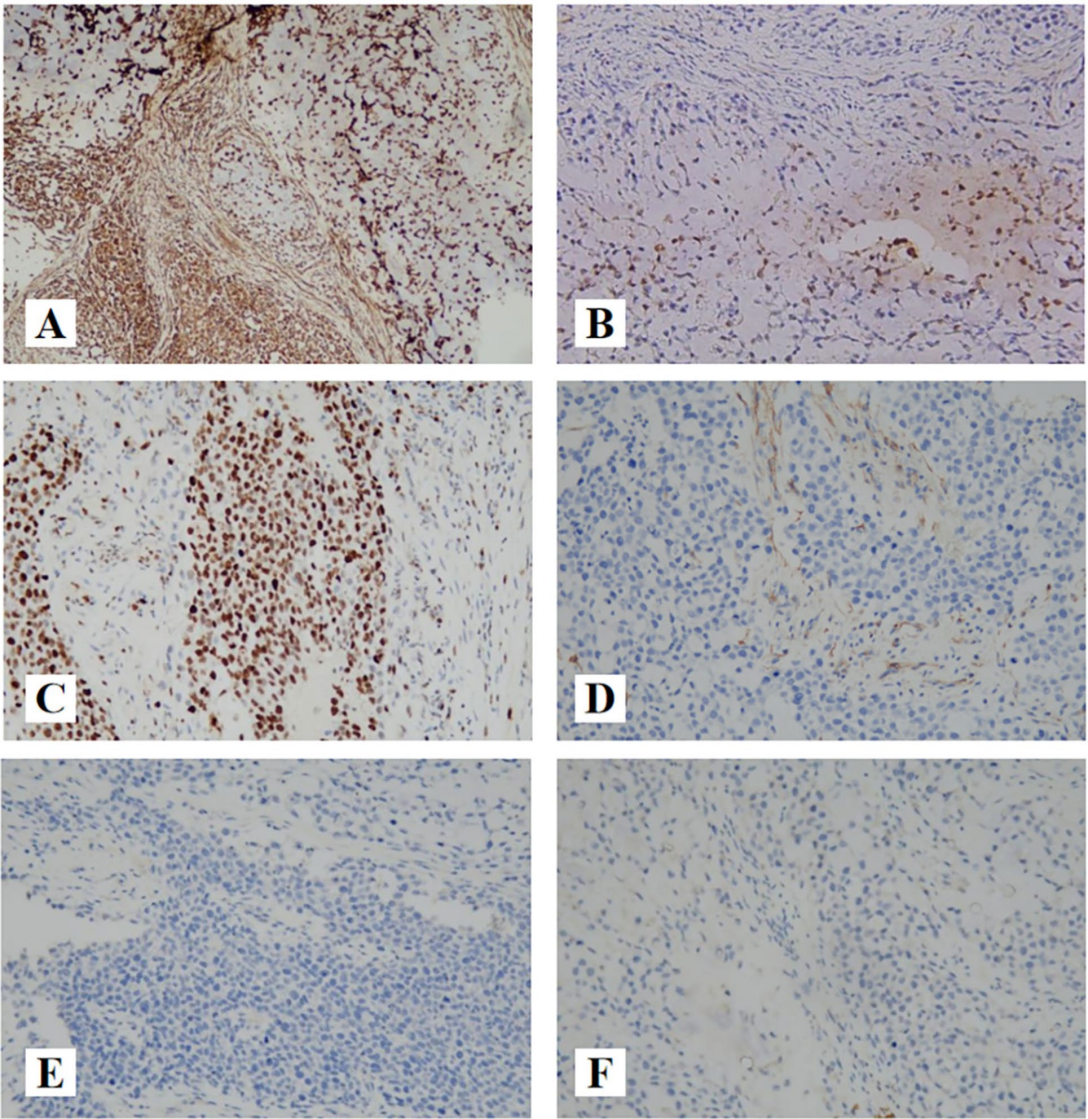
The immunoprofile of EMC. **A** (IHC × 100): Vimentin showing positive immunoreactivity in the cytoplasm. **B** (IHC × 200): S-100 showing focally positive immunoreactivity in the cytoplasm and nucleus. **C** (IHC × 200): Nuclear staining for Ki-67 with index labeling of 80%. **D** (IHC × 200): Smooth muscle actin (SMA), negative. **E** (IHC × 200): KRT7, negative. F (IHC × 200): SOX10, negative

The immunohistochemical markers showed positive staining for vimentin, focally positive for P63, KRT14, S-100, Ki-67(80%) and showed negative staining for KRT5/6, KRT7, GFAP, cal-ponin, SMA, KRT-PAN, SOX10, mammaglobin (Fig. 3).

**Fig. 4:**
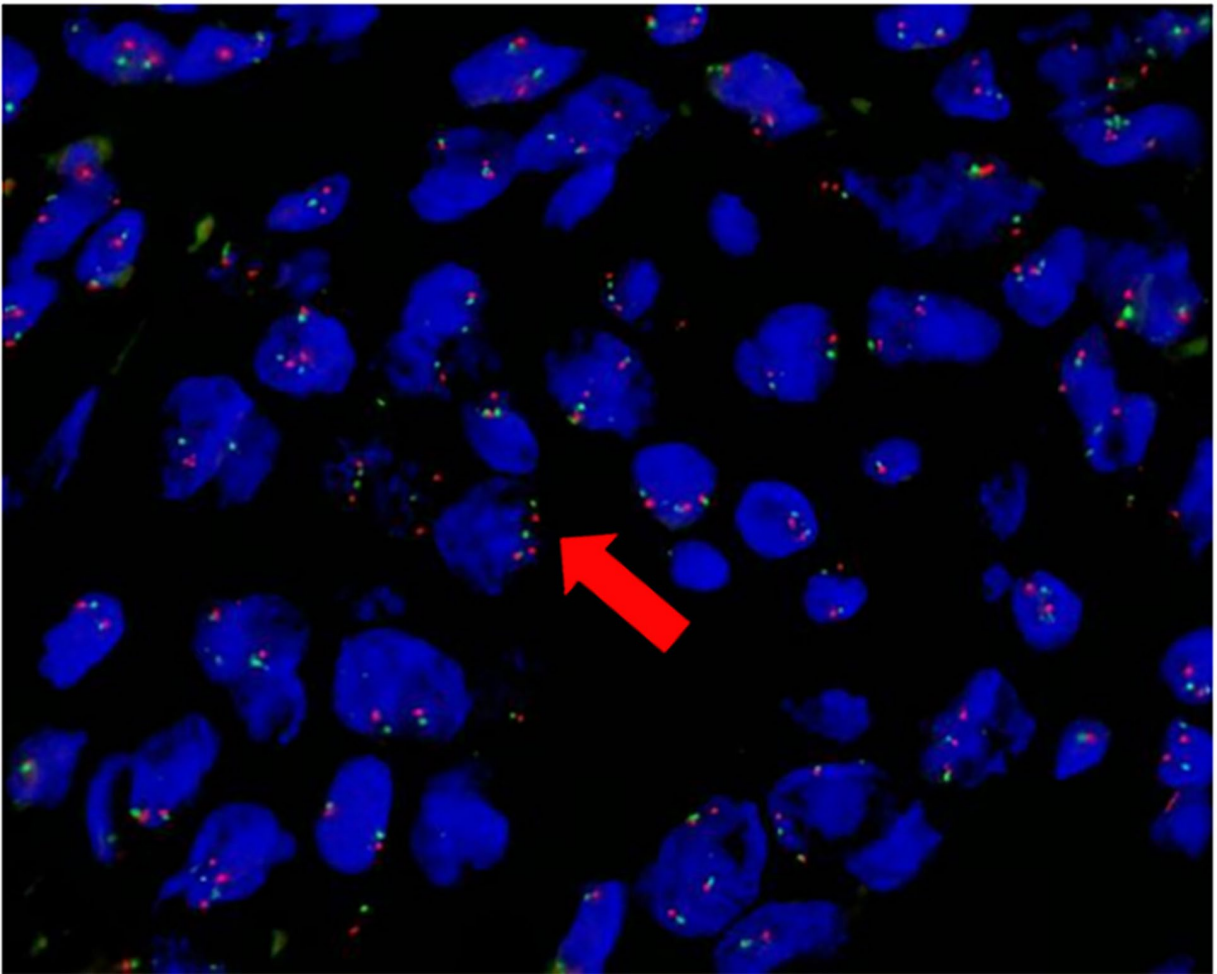
FISH images of the tumor. Orange (R) signal marks the 3 'end of NR4A3 gene, green (G) signal marks the 5 'end of NR4A3 gene. The normal signal mode is 2F (F is the yellow signal of red-green fusion), and the typical positive signal mode is 1F1R1G. FISH analysis on the histological section evidenced rearrangement of NR4A3 gene(arrow)

FastProbe NR4A3 (9q22) gene break two-color probe was used for FISH detection, and in this case FISH analysis showed rearrangement of NR4A3 (Fig. 4).

A final diagnosis was made as EMC combining clinical examination, pathological characteristics, IHC and FISH results.

## Discussion

**Fig. 5:**
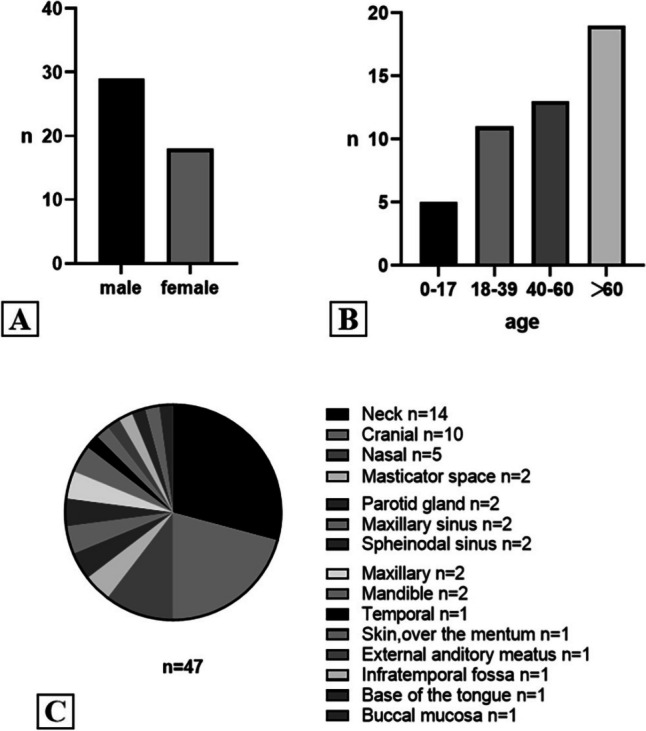
Epidemiological findings of EMC. **A** Male–female ratio of EMC patients. **B** Age distribution of EMC patients. **C** The distribution of the primary lesion sites in the head and neck region

EMC has been classified as a type of soft tissue tumor with uncertain differentiation according to the World Health Organization Classification of Tumors of Soft Tissue and Bone [5]. Approximately 80% of the cases occur in the deep soft tissues of the proximal extremities and limb girdles and most commonly involve the thigh, which accounts for about 69% of the cases. Approximately 20% of the cases are located in the trunk, mainly in skeletal muscles, tendons and the deep subcutis; a few cases involve the skin and bone tissue, whereas rare sites include the vulva and the breast [5, 6]. After we retrospect 48 cases from 1981–2022 which took place in the head and neck region [1, 7], we found that males account for 60.4%(*n* = 29), while females make up 37.5%(*n* = 18), and 2.1%(*n* = 1 case) was unavailable, the male-to-female ratio of about 1.5:1 (Fig. 5A). Most people were affected after their fourth decade of life (Fig. 5B). In the head and neck region, the common sites of EMC includes the nasal cavity, neck as well as intracranial (Fig. 5C). The pathogenesis of EMC remains controversial. Surgical or accidental trauma may be key factors, along with the inhalation of chemical carcinogens such as hydrocarbons [1, 7]. Our patient did not report any intraoral region trauma. Clinical examination of EMC has no specific findings which separates it from other types of chondrosarcomas. Pain, tenderness, and detection of a palpable mass may characterize some cases [8]. The clinical behaviour of this tumour may be indolent or aggressive, depending on the grade. Local recurrence, distal metastases or both may be present during the course of the disease. Distal metastases have been recorded in lungs, soft tissues, bones, regional lymph nodes, subcutis, brain, bones and testis. Recurrence and metastases after long intervals are also known to occur [9].

Histologically, EMC usually exhibits a nodular structure with abundant myxoid stroma [5]. homogeneous stroma, primarily hyalinized and focally chondroid, predominated. Within the stroma, epithelial cells, arranged in strands and small islands, were widely dispersed; cellular atypia and infiltrative growth were identified. Areas of atypical cartilage with hyperchromatic and occasionally binucleated cells were seen. However, there appeared to be very few necrosis, and the mitotic figures are 2-3̸10 high-power fields (HPFs) [5, 10, 11]. Immunohistochemistry plays a significant role in EMC diagnosis. In the present case, IHC examination of the tumor revealed that vimentin was positively stained, P63, KRT14, S-100 was focally positive, Ki-67(80%), while KRT5/6, KRT7, GFAP, calponin, SMA, KRT-PAN, SOX10, mammaglobin was negative. FISH detection plays an increasingly important role in diagnosing EMC. Rearrangement of NR4A3 has been found exclusively in EMC and is considered a hallmark of EMC according to the WHO [12]. In EMC, the major fusion partners of NR4A3 described so far belong to TET family genes: EWSR1 (over 70%), TAF15 (about 20% of cases), and FUS. Rarer NR4A3 fusion partners (< 5%) include transcription factors TCF12 and TFG [13]. Due to its rarity in this area, the differential diagnosis from other tumors should be paid attention to.

**Fig. 6:**
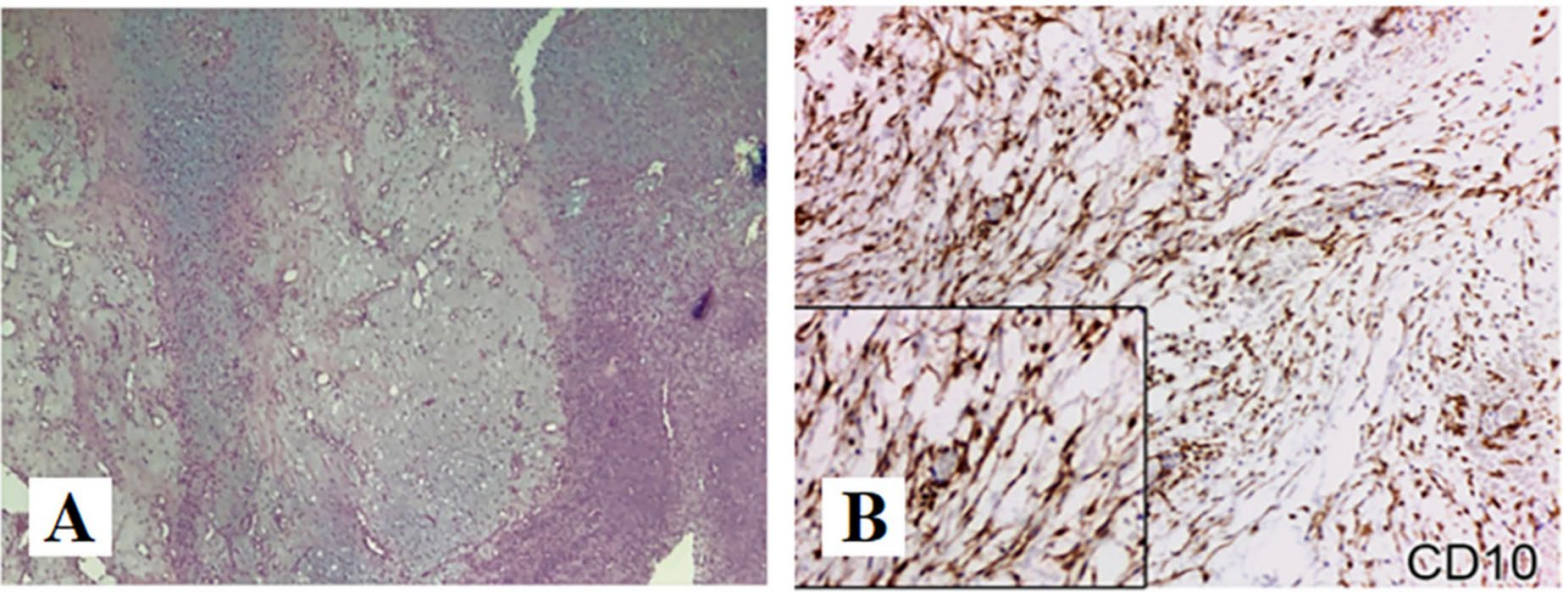
**A** (HE × 40): Lobules of uniform spindle or stellate cells with myxoid or chondroid to fibrous stroma of CMF. **B** (IHC × 200): CD10, positive [17, 18]

Chondromyxoid fibroma(CMF). CMF is a benign bone tumor originating from cartilage tissue, which is more commonly seen in the metaphysis of long bones. CMF mainly affects the second and third decade of young adults [14]. Around 80% of patients are < 36 years. The tumor is not gender specific and both males and females are affected equally; however, some series showed a slight male predominance [15]. The pathological characteristics of CMF includes lobules of uniform spindle or stellate cells with myxoid or chondroid to fibrous stroma and intermittently seen multinucleated giant cells, with calcifications being more prevalent in craniofacial lesions than in peripheral locations [14] (Fig. 6A). Radiographic findings show well-defined tumors with sclerotic rims and scalloped margins; intra-tumor calcification; low signal intensity on T1-weighted images and heterogeneous high signal intensity on T2-weighted images [16]. In the respect of immunohistochemistry, a positive expression of CD10, which can help in the diagnostic process [17] (Fig. 6B).

**Fig. 7:**
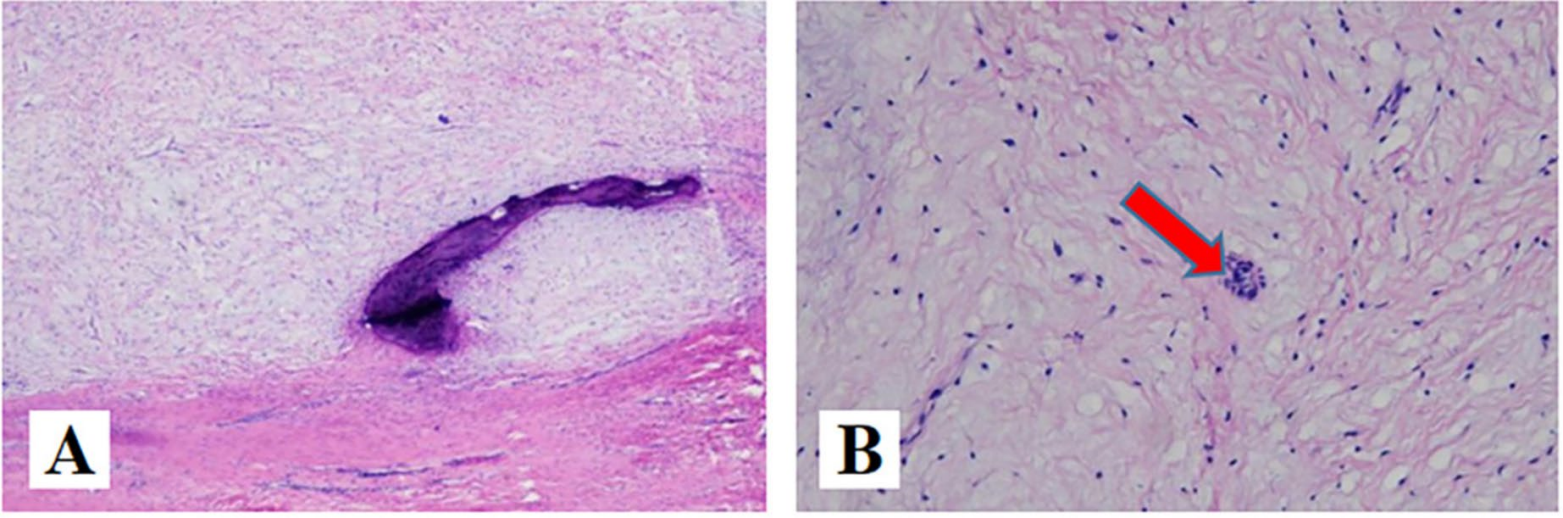
**A** (HE × 40): stellate and spindle-shaped cells embedded in a richly myxoid extracellular matrix. **B** (HE × 200): Residual odontogenic epithelium can be seen(arrow)

Odontogenic myxomas(OM). OM are rare benign tumors of mesenchymal origin. It most frequently occurs in the second to fifth decade of life and the average age of occurrence ranges from 23 to 30 years. Women are more commonly affected than men with a ratio of 1.5:1 [19, 20]. The mandible is the more commonly affected than maxilla, with the posterior body, ramus, and angle being the most common sites, respectively [21]. Regardless of the jaw, odontogenic myxoma is usually found in relation to a tooth, typically a premolar or molar [22]. Microscopically these lesions are characterized by stellate and spindle-shaped cells embedded in a richly myxoid extracellular matrix (Fig. 7A), islands of inactive odontogenic epithelium may be found in a few cases [23] (Fig. 7B). KRT19 positivity observed in some of cases confirm the occurrence of odontogenic epithelial remnants [24], which may be in favor of the diagnosis.

**Fig. 8:**
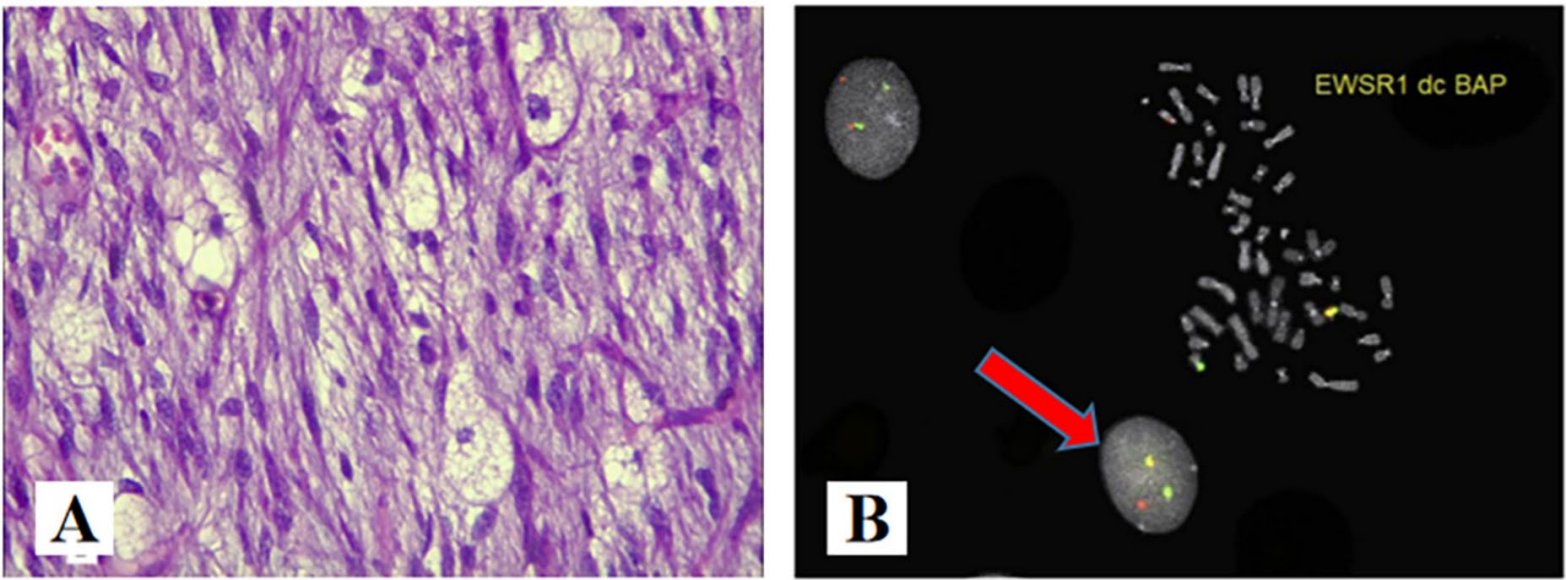
**A** (HE × 400): Abundant myxoid stroma, proliferation of lipoblasts in variable numbers and fine vascularization, A network of chicken's foot like capillaries can be seen in the mesenchyma. **B** Cytogenetic and molecular cytogenetic findings in a myxoid liposarcoma with cryptic EWSR1-DDIT3 fusion(arrow). Metaphase fluorescence in situ hybridization (FISH) with a break-apart probe specific for the EWSR1 gene. An intact yellow signal is seen on the normal chromosome 22, whereas a split signal is seen on the derivative chromosomes 11 (green) and 22 (red) [26, 27]

Myxoid Liposarcoma(MLPS). Out of all liposarcomas, MLPSs occur in adolescents and young adults. It shows predilection for thigh [25]. Chicken-wire capillary vasculature is characteristic of this tumor. Cells with cytoplasmic vacuolations with eccentric appearing nuclei are also seen (Fig. 8A). Few cases with discrete cartilaginous, leiomyomatous and osseous differentiation in MLPS [25]. Genetic molecular studies may play a significant role in identifying the tumor type. Chromosomal abnormality such as FUS-DDIT3 causes chromosomal translocation abnormality namely t(12;16)(q13;p11), and chromosomal abnormality such as EWSR1-DDIT3 causes chromosomal translocation abnormality namely t(12;22)(q13;q12) [26] (Fig. 8B).

**Fig. 9:**
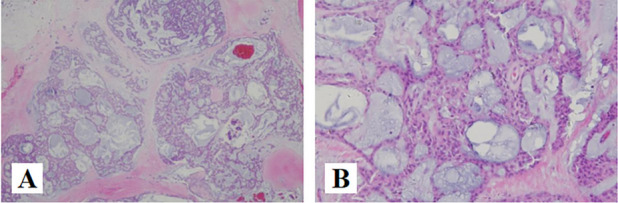
**A** (HE × 40): Architecturally, the tumor cells are arranged into nests and a large number of mucus-like areas can be seen. **B** (HE × 100): The tumor cells are plasmacytoid cells

Myoepithelial carcinoma(MEC). MEC usually occur in young to middle-aged adults in the extremities and limb girdles, it tends to be identified more in the females. It is more frequently reported in major salivary glands, especially the parotid, it may also occur in the small salivary glands of the nasal cavity and palate. Architecturally, tumor cells may be arranged in solid, trabecular, cribriform, thin cords, small clusters, and tubular formations, showing a multinodular, lobulated, or sheet-like appearance [28] (Fig. 9A). Histologically, myoepithelial carcinoma is rather heterogeneous, presenting a variety of cell types, including epithelioid, spindle, basaloid, plasmacytoid, and clear cells. Despite this, one cell type is usually predominant for each tumor (Fig. 9B). Cytology is typically bland, and atypia is not a prerequisite for malignancy in myoepithelial carcinoma. However, some tumors might present high-grade features, such as tumor necrosis and increased mitoses.

**Fig. 10:**
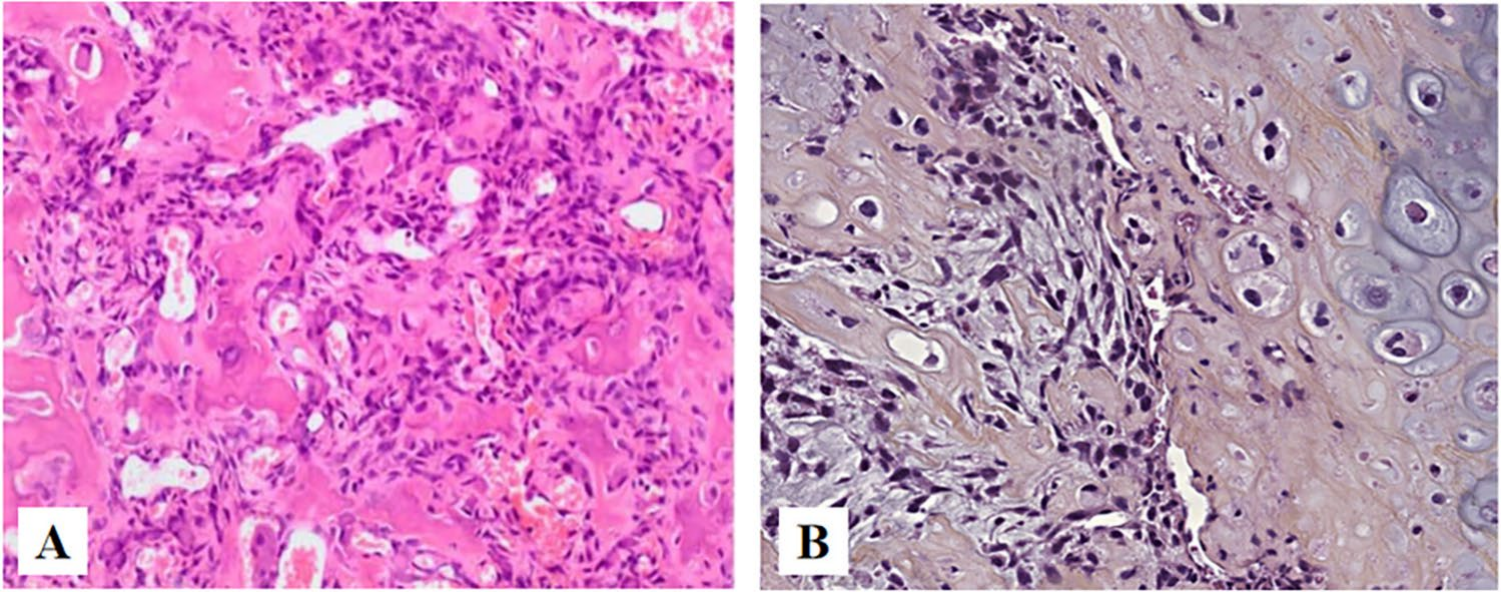
**A** Interconnecting trabeculae of woven bone rimmed by plump osteoblasts and richly vascularized fibroblastic stroma are present. **B** Substantial chondroblastic differentiation and nuclear atypia [33, 34]

Osteosarcoma(OST). OST mainly affects the metaphyseal growth plates in the long bones. OST of the jaw(OSJ) is rare, comprising of only 6% to 7% of all OST and 1% of all head and neck malignant neoplasms [29]. OST often affects patients in their first two decades of life whereas OSJ tends to be diagnosed two decades later, show better survival rates. OSJ affects men and women in equal proportions and is slightly more common in the mandible [30]. Predisposing factors include Paget disease, Li-Fraumeni syndrome, or other intraosseous diseases, such as fibrous dysplasia or cemento-ossifying fibroma. Radiation to the head and neck area has been reported in multiple large studies, and now is considered to play a role in approximately 10% of all OSJ cases [31], while there were no obvious causes in this case. Patients with OSJ present with swelling in contrast to “pain during activity” in OST. The typical morphology is of malignant spindle and polygonal cells forming neoplastic osteoid with an interlaced and irregular deposition. Mitotic activity and permeative growth into surrounding bone are usually apparent (Fig. 10) [32].

**Fig. 11:**
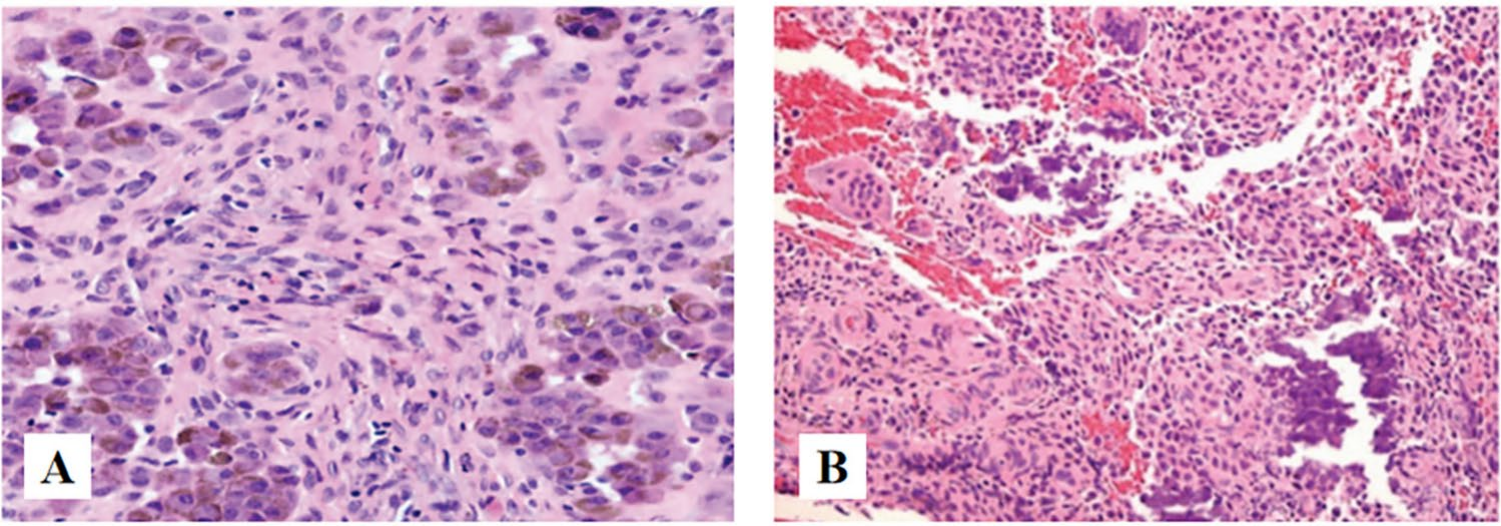
**A** (HE × 40): Chondrocytes with deposition of hemosiderin deposition. **B** (HE × 40): The calcifications present as ‘‘chicken-wire’’ appearance [37]

Chondroblastoma. Chondroblastoma is a benign, cartilage producing neoplasm. It most commonly affects the epiphyses of long bones. Craniofacial bones especially the temporal bones, are its favored site of occurrence, with the incidence ranging from 1%-7% of all chondroblastoma. It mostly affects patients in their first 2 decades of life [35]. There is a male preponderance with a male-to-female ratio of 2 to 1. The most common presenting symptoms include hearing loss, otalgia, tinnitus, aural fullness, and vertigo or disequilibrium [36]. Chondroblastoma is characterized histologically by a sheetlike proliferation of small to intermediate-sized round polygonal cells. In addition to the above, variable numbers of multinucleated giant cells are often presented, as are foci of hemosiderin deposition. The latter occurs more commonly in the tumors located in the skull and facial bones. In most lesions, islands of mature cartilaginous differentiation can be found, containing foci of eosinophilic chondroid matrix (Fig. 11A). Matrix formation must be seen to confirm a diagnosis of Chondroblastoma. Mitoses are occasionally found, with an average count of 1 to 3 mitotic figures per 10 HPF. Atypical mitotic figures should not be seen, and if present, tend to exclude Chondroblastoma from the differential diagnosis (Fig. 11B) [37].

**Fig. 12:**
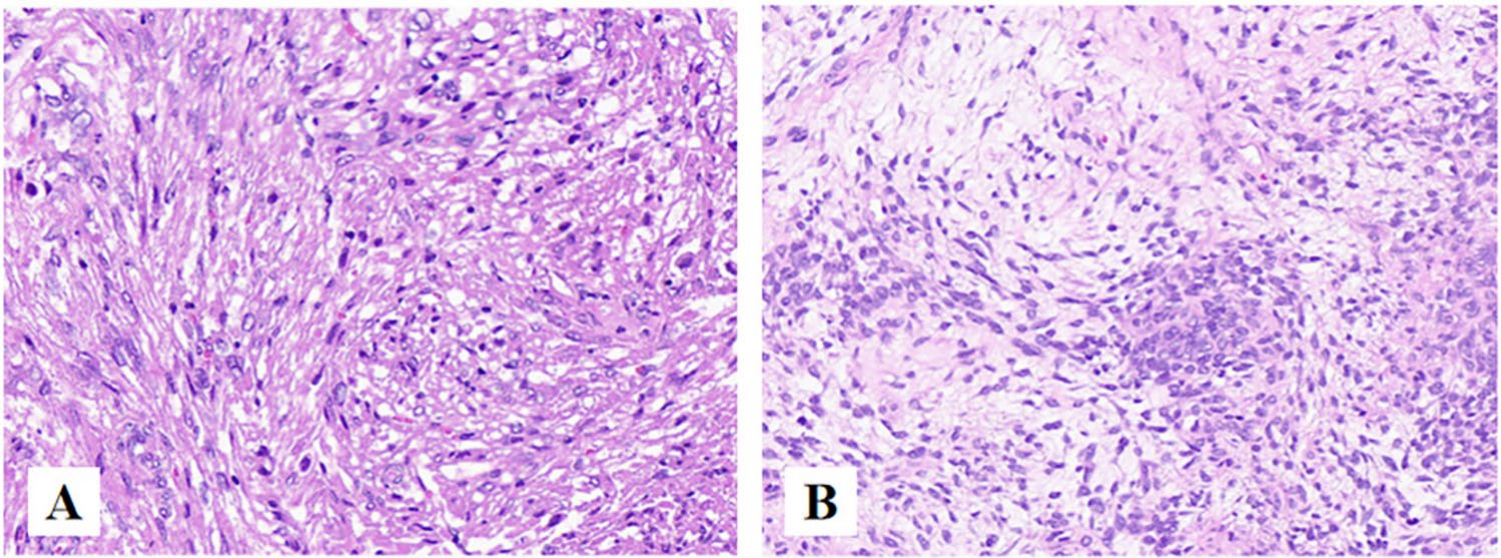
**A** Monophasic SS, made of spindle cells with moderate cytologic atypia and differentiated areas of variable cellularity. **B** Biphasic SS composed by glandular-like structures with spindle cells [38]

Synovial sarcoma(OST). Synovial sarcoma (SS) is a soft tissue malignant tumor. It primarily affects people in their thirties and most frequently occurs in the extremities (70%), followed by the trunk (15%), and least common in the head and neck region (5%–7%), the most common site of HNSS is the hypopharynx [38]. It primarily affects young adults in their thirties. SS is divided into three distinct histologic subtypes: monophasic SS, biphasic SS, and poorly differentiated SS. Monophasic SS contains uniform spindle cells, biphasic SS consists of epithelial cells arranged into glandular structures with spindle cells arranged into fascicles, and spindles and round blue cells characterize poorly differentiated SS (Fig. 12) [38]. Notably, Immunohistochemistry plays a crucial role in diagnosis, SS is positive for epithelial markers, including cytokeratin, epithelial membrane antigen (EMA), and vimentin. SS is usually unfavorable for CD34 and FLI-1 [39].

Immunohistochemistry is an important aid in the diagnosis of this tumor. Neoplastic myoepithelial cells might show a variable staining pattern. Therefore, different myoepithelial markers should be analyzed, in order to confirm the origin when considering this diagnosis. Myoepithelial carcinoma usually stains positive for high molecular weight cytokeratins (AE1/AE3, KRT14) and myoepithelial markers, such as smooth muscle actin (SMA), p63, S100, GFAP, and calponin. Most tumors are negative for CEA, KRT7, and EMA. FISH and/or gene rearrangement studies offer additional useful information in rendering an accurate diagnosis, the identification of a EWSR1 gene rearrangement or EWSR1 gene fusion may assist in the diagnosis of MEC [40].

## Conclusion

EMC is a rare entity which take place in the head and neck region, as for the intral-oral area such as the gingival present here, is extremely infrequent. Due to its scarcity of the intraoral area and the morphology mimicking of other mesenchymal origined neoplasms, the correct diagnosis of EMC is a great challenge for pathologists. Traditional single microscopic histology is considered to be the gold standard for the diagnosis of tumors. However, in this case, by a combination of histological findings, immunohistochemistry, and molecular pathology, a definitive diagnosis was made as EMC. After all, IHC and FISH have necessity to be taken more consideration into the diagnosis of tumors as long as the conditions acquired to gain more precision.

## Data Availability

Not applicable.

## Abbreviations

EMC: Extraskeletal Myxoid Chondrosarcoma
IHC: Immunohistochemistry
FISH: Fluorescent in situ Hybridization
CT: Computed Tomography
CBCT: Cone Beam Computed Tomography
CMF: Chondromyxoid fibroma
OM: Odontogenic myxomas
MLPS: Myxoid liposarcoma
MEC: Myoepithelial carcinoma
OST: Osteosarcoma
SS: Synovial sarcoma
